# Excess use of thyroid hormone treatment among patients with fibromyalgia: a cross-sectional study in primary health care

**DOI:** 10.1186/s13104-022-05971-y

**Published:** 2022-02-24

**Authors:** Varinen Aleksi, Kosunen Elise, Tuomas H. Koskela

**Affiliations:** 1 Department of General Practice, Faculty of Medicine and Health Technology, Tampere University, Arvo-Building B222, PL100, 33014 Tampere, Finland; 2 Centre for General Practice, Tampere University Hospital, Tampere, Finland

**Keywords:** Fibromyalgia, Thyroid hormone treatment, Family practice, Drug prescription, Cross-sectional study

## Abstract

**Objective:**

From previous studies, it is known that the association between fibromyalgia and thyroid autoimmunity diseases exists. On the other hand, it was recently suggested that in many cases thyroid hormone treatment might be unnecessary. The aim of our study is to explore the thyroid hormone treatment among primary health care fibromyalgia patients. Our study is cross-sectional and based on fibromyalgia study from the city of Nokia Health Center. Clinical examination was performed to participants, patients filled five questionnaires and information from electronic patient records was gathered. In addition to other parameters, we studied patient’s thyroid function levels at the beginning of thyroid hormone treatment.

**Results:**

From those patients participating in the study (n = 103), 34% (n = 33) had thyroid hormone treatment. From those taking thyroid hormone treatment, 48% (n = 16) had information regarding the initial TSH and T4-V levels at the beginning of the treatment. 37% (n = 6) of them had normal thyroid function. Small sample size and data gathered from single health center effects on the generalizability of our findings. However, we suggest further studies to confirm the potential association between fibromyalgia and inappropriate thyroid hormone treatment.

## Introduction

Fibromyalgia is a functional syndrome characterized by disturbances of sympathetic nervous system and central sensitization [1]. Although fibromyalgia is not regarded as an autoimmune disease, several autoimmune diseases—like thyroid autoimmunity—are associated with it [2]. In a Japanese study, 7.7% of fibromyalgia patients had hypothyroidism, but no association between fibromyalgia symptom severity and anti-thyroid peroxidase (TPO) antibodies was found [3].

The incidence of overt hypothyroidism is reported to be stable around 0.2–2.0% of population. In Finland, the prevalence of hypothyroidism was 3.6% in 2007 based on regularly purchased levothyroxine sodium tablets [4]. However, subclinical hypothyroidism affects up to 12% of population and the use of levothyroxine is increasing. [5] It was recently suggested, that in Finland every third levothyroxine-treatment might be unnecessary, as the number of patients taking levothyroxine has almost doubled during last 10 years, although the prevalence of hypothyroidism has not changed [6]. The levothyroxine treatments have increased also in USA, UK and Sweden, where the prevalence of subclinical hypothyroidism is estimated to be around 12–18% [7].

The symptoms of hypothyroidism are diverse, and the diagnosis of hypothyroidism should be based on laboratory tests, because none of the symptoms or clinical signs are sensitive or specific enough [8]. Around 20–25% of patients with normal thyroid function present with one or two classic hypothyroidism symptoms such as constipation, fatigue, muscle weakness, dry skin, memory difficulties and feeling cold [9].

In clinical practice, the diagnosis of fibromyalgia should be based on multifocal pain not explained by injury or inflammation. Other symptoms include fatigue, memory difficulties, sleep disturbances, irritable bowel symptoms and mood problems [1]. Many of these symptoms overlap with hypothyroidism. Consequently, it is suggested that other disorders that commonly present with fatigue, such as anemia and thyroid disease, are ruled out when the diagnosis of fibromyalgia is set [10]. On the other hand, it is known that patients with neuropsychiatric symptoms may attribute unrelated symptoms to thyroid dysfunction and this can lead to overtreatment [11].

In conclusion, it is known that thyroid autoimmunity associated to fibromyalgia and the symptoms of these two conditions overlap considerably. The number of levothyroxine treatments is increasing even though the incidence of overt hypothyroidism is reported to be stable. According to guidelines, the diagnosis of hypothyroidism should be based on the findings from laboratory tests. To the best of the author’s knowledge, there are no previous studies addressing the association between functional syndromes, such as fibromyalgia, and inappropriate levothyroxine use.

This study is based on data from patients with fibromyalgia participating in the Finnish Primary Health Care study conducted at Nokia Health Centre and the objective of that study is to describe the demographic features of patients with fibromyalgia in a Finnish health center.

Our aim is also to explore the occurrence of thyroid hormone treatment among fibromyalgia patients and thyroid hormone levels at the beginning of their treatment and to evaluate were the guidelines of care followed.

## Main text

### Materials and methods

Population of the city of Nokia was 33,210 in 2016 and 19 doctors were working in the Nokia Health Center at the time of the study.

Fibromyalgia patients were searched from the electronic patient records of the health center. Inclusion criteria were ICD-10 code corresponding to fibromyalgia (M79.7) or diagnosis of fibromyalgia with some other codes (M79.0, M25.5, R52.9 and M79.1). Altogether 208 fibromyalgia patients were identified. Information letter containing five questionnaires, including Fibromyalgia Impact Questionnaire (FIQ) and Patient Health Questionnaire-9 (PHQ-9), were sent to the patients. GP’s appointment was scheduled for the patients responding to the letter. Functional ability was derived from FIQ and depression from PHQ-9. American College of Rheumatology (ACR) 2010 diagnostic criteria form was filled with the patients at the beginning of consultation. Only the patients filling the criteria for fibromyalgia were included in the study.

Information from thyroid hormone treatment and TSH and free thyroxine (T4-V) levels were gained from the electronic patient record. Reference values for TSH and T4-V were provided by the Fimlab Laboratories, which provides laboratory services to the Tampere University Hospital area. We used the Finnish guidelines for hypothyroidism [12]: TSH level over 4.2 mU/l was defined as subclinical hypothyroidism if the T4-V level was normal. The T4-V level under 11.0 pmol/l was defined as central hypothyroidism if TSH level was normal or low. TSH level over 4.2 mU/l and T4-V level under 11.0 pmol/l was defined as overt hypothyroidism. TSH levels between 0.27 and 4.2 mU/l and T4-V levels between 11.0 and 22.0 pmol/l were defined as normal thyroid function.

Data were analyzed using SPSS version 23. Cross-tabulation and Chi-square test were used when categorical variables were present and two-sample t-test was performed with variables following a normal distribution. Paired samples t-test was used when the means of two measurements taken from the same individual was compared. When needed, Kolmogorov–Smirnov test was used to confirm normal distribution of variables.

The Regional Ethics Committee of Tampere University Hospital has approved the study plan.

### Results

#### Demographic features of study population

Altogether 208 fibromyalgia patients (17 male and 191 female) were included in the study. 103 patients returned mailed questionnaires and GP’s appointment was scheduled. There was no statistically significant difference between those not answering the study invitation and those participating in the study regarding age, gender, number of regularly taken medicines, GP’s visits before the study and number of other diseases (Table 1).

**Table 1:** Features of study population

	Participants N = 103		Not participating N = 105		p-value
	%	n	%	n	
Gender					0.19
Male	10.7	11	5.7	6	
Female	89.3	92	94.3	99	

	Mean and 95% CI for mean				
Age	55.1 (52.1–58.1)		57.4 (54.4–60.5)		0.30
Number of regularly taken medicines	3.3 (2.7–3.4)		4.0 (3.3–4.7)		0.15
GP’s visit 12 months before the study	5.3 (4.0–6.5)		5.0 (4.0–6.0)		0.52
Number of other chronic diseases	2.5 (2.2–2.9)		2.8 (2.4–3.2)		0.39

#### Features of patients with levothyroxine treatment

Altogether 96 patients had fibromyalgia according to ACR 2010 criteria and seven patients did not fill the criteria for fibromyalgia at the time of the study. From that group 33 (34%) had thyroid hormone treatment and 63 (66%) had not. No statistical significant difference was found between those taking thyroid hormone replacement and those not taking it in functional ability (p = 0.36) or depression (p = 0.71). Information regarding regular medication was present in their electronic patient record (Table 2).

**Table 2:** Characteristics of study population

	No levothyroxine	Levothyroxine	p-value
	N = 64	N = 33	
	Mean and 95% CI for mean		
Number of other diseases	2.4 (1.91–2.90)	2.79 (2.32–3.25)	0.32
Number of regularly taken medicines	3.05 (2.15–3.95)	3.45 (2.63–4.28)	0.65
FIQ score (mean)	44.42 (39.87–48.97)	41.07 (34.36–47.77)	0.36
PHQ-9 score	10.05 (8.64–11.46)	10.52 (8.39–12.64)	0.71

All characteristics were normally distributed

*FIQ*  Fibromyalgia Impact Questionnaire, *PHQ-9 * Patient Health Questionnaire for depression

#### Thyroid hormone levels before and after treatment

From those 33 patients with levothyroxine treatment, 16 had information regarding the initial TSH and T4-V levels before thyroid hormone treatment: ten (10/16) patients had hypothyroidism based on the laboratory tests. Subclinical hypothyroidism was present in six cases (6/16), central hypothyroidism in three cases (3/16) and overt hypothyroidism in one case (1/16). The diagnosis of central hypothyroidism was confirmed with specialist of internal medicine working in the Nokia Health Center in all three cases. Of the 16 patients with thyroid hormone treatment, six patients (37%) had normal thyroid function at the beginning of the treatment (Table 3).

**Table 3:** Thyroid function of patients (n = 16) with levothyroxine treatment whose initial TSH and T4-V level information was present in patient records

Patient	Daily thyroxine dose (μg)	TSH (mU/l) before threatment	T4-V (pmol/l) before treatment	Current TSH (mU/l)	Current T4-V (pmol/l)	Year of hypothyroidism, dg
1 overt hypothyroidism	75	7.3	10.0	2.5	13.6	2012
2 subclinical hypothyroidism	37.5	7.9	11.2	1.4	15.1	2012
3 subclinical hypothyroidism	93	7.1	11.0	0.31	19.1	2008
4 subclinical hypothyroidism	75	6.7	13.8	3.2	15.7	2004
5 subclinical hypothyroidism	50	5.7	13.0	2.5	16.0	2016
6 subclinical hypothyroidism	50	4.9	13.7	2.6	18.6	2011
7 subclinical hypothyroidism	50	4.4	14.1	2.8	14.1	2014
8 central hypothyroidism	75	2.6	10.8	0.78	19.3	2012
9 central hypothyroidism	125	2.6	10.7	1.3	11.6	2012
10 central hypothyroidism	71	0.24	10.9	0.18	14.2	2011
11 normal thyroid function	50	3.5	12.1	1.6	16.2	2013
12 normal thyroid function	100	1.6	16.6	1.2	16.0	2015
13 normal thyroid function	50	1.9	12.6	1.3	13.6	2013
14 normal thyroid function	150	4.2	11.9	0.01	20.2	2012
15 normal thyroid function	100	1.2	13.9	0.87	20.3	2014
16 normal thyroid function	25	1.1	11.0	0.87	13.9	2016
Mean (p-value)	73.5	3.9	12.3	1.5 (< 0.001)	16.1 (< 0.001)	
Median	73	3.83	12	1.3	15.85	

Overt hypothyroidism (TSH > 4.2 mU/l and T4-V < 11.0 pmol/l), subclinical hypothyroidism (TSH > 4.2 mU/l and T4-V = 11.0–22.0 pmol/l), central hypothyroidism (TSH = 0.27–4.2 mU/l and T4-V < 11.0 pmol/l), normal thyroid function (TSH = 0.27–4.2 mU/l and T4-V = 11.0–22.0 pmol/l). Paired samples T-test was used to determine the statistical significance for chance of TSH values and T4-V values

### Discussion

In our study, over one-third of the fibromyalgia patient using thyroid hormone treatment—whose initial thyroid hormone levels were available—did have normal thyroid function. This indicates that the guidelines of care are not followed in one-third of the cases when levothyroxine treatment is prescribed to a patient presenting fibromyalgia symptoms.

The occurrence of thyroid hormone treatment was much higher in our study population (34%) than the known prevalence of hypothyroidism among general population in Finland [4].

There are likely several explanations to the relatively weak adherence of the guidelines of care for treatment of hypothyroidism with fibromyalgia patients. One might be that one of the main symptoms of fibromyalgia is fatigue, which also is common with hypothyroidism. Patients are aware of this and some may want to try out the thyroid hormone treatment even though their thyroid function is normal. On the other hand, it might be tempting for a physician to diagnose a somatic disease—instead of a functional syndrome—causing the fatigue and try out if levothyroxine treatment would reduce patient’s symptoms. Altogether, further studies are needed to study these hypotheses.

In our study, three patients had central hypothyroidism and only one patient had overt hypothyroidism. These patients have obvious indication for the treatment. Rest of the patients (12 patients) had either subclinical hypothyroidism (6 patients) or normal thyroid function (6 patients) at the beginning of the treatment. These patients might not benefit from levothyroxine treatment.

Our findings that 37% of patients had normal thyroid hormone levels at the beginning of the treatment is consistent with the suggestion that every third levothyroxine treatment might be unnecessary in Finland [6]. On the other hand, it is probable that subclinical hypothyroidism is diagnosed more often from fibromyalgia patients than from general population, because guidelines give instructions to test thyroid function when fibromyalgia diagnosis is set [10]. Based on literature, it is unknown whether patients benefit from levothyroxine treatment in that case [13, 14].

In addition, the mean daily dose of levothyroxine was 73.5 μg in our study, which is lower than 92.6 μg described by Virta and Eskelinen in their study [3]. In our study, seven patients (44%) had a low daily dose of levothyroxine (< 51 μg) comparing to 13.9% in Finnish prevalence study 1. Furthermore, we found no association between thyroid hormone treatment and functional ability or depression. This finding is consistent with previous studies [3].

Even though fibromyalgia is not regarded as an autoimmune disease, several autoimmune diseases are associated with it [2]. There is evidence that autoimmune diseases such as type 1 diabetes, multiple sclerosis and Hashimoto’s thyroiditis could be derived from pathogens like *Mycobacterium paratuberculosis* [15, 16]. Furthermore, inflammatory, infectious, and autoimmune disorders have also been suggested to be etiological events in the development of fibromyalgia, but there is limited evidence to support that hypothesis [17].

To conclude, our findings suggest that there is considerable unnecessary prescribing of levothyroxine to patients with fibromyalgia syndrome with normal thyroid function. However, small sample size and data gathered from a single health center effects on the generalizability of our findings. Further studies are needed to confirm the potential association between functional syndromes like fibromyalgia and inappropriate thyroid hormone treatment. Moreover, it is not clear do fibromyalgia patients benefit from diagnosis of subclinical hypothyroidism, which might be an incidental finding from thyroid function screening at the time of the diagnosis of fibromyalgia. Further studies are also needed from that subject.

## Limitations

The main weakness of our study is small sample size. Furthermore, data were collected only from one health center, which has an impact on the generalizability of our findings. In addition, we do not know for sure what kind of symptoms patients had at the moment of the diagnosis of thyroid dysfunction, but we know from the fibromyalgia questionnaires that all patients experienced symptoms that are also linked to hypothyroidism—such as fatigue—at the moment of the study. Another weakness is that only half of patients identified from patient records participated in the study. On the other hand, there was no statistically significant difference between participants and those not participating (Table 1).

Furthermore, altogether 33 patients had levothyroxine treatment, but we had information only from 16 patients’ thyroid function before the treatment. It is likely, that patients without this information, either had had levothyroxine treatment for a long time or it was prescribed from occupational health care or from specialized health care using different electronic patient records. For this reason, we believe that these 16 patients are rather representative sample of fibromyalgia patients with levothyroxine treatment in a Finnish health center.

## Data Availability

The data that support the findings of this study are available from Nokia Health Center but restrictions apply to the availability of these data, which were used under license for the current study, and so are not publicly available. Data are however available from the authors upon reasonable request and with permission of Nokia Health Center.

## Abbreviations

ACR: American College of Rheumatology
FIQ: Fibromyalgia Impact Questionnaire
GP: General practitioner
PHQ-9: Patient Health Questionnaire-9
T4-V: Free thyroxine
TPO: Anti-thyroid peroxidase
TSH: Thyroid-stimulating hormone
